# Higher-Order Chromatin Regulation of Inflammatory Gene Expression

**DOI:** 10.1155/2017/7848591

**Published:** 2017-04-09

**Authors:** Jin-Wen Xu, Shuang Ling, Jun Liu

**Affiliations:** Institute of Interdisciplinary Research Complex, Shanghai University of Traditional Chinese Medicine, Shanghai 201203, China

## Abstract

Whether it is caused by viruses and bacteria infection, or low-grade chronic inflammation of atherosclerosis and cellular senescence, the transcription factor (TF) NF-*κ*B plays a central role in the inducible expression of inflammatory genes. Accumulated evidence has indicated that the chromatin environment is the main determinant of TF binding in gene expression regulation, including the stimulus-responsive NF-*κ*B. Dynamic changes in intra- and interchromosomes are the key regulatory mechanisms promoting the binding of TFs. When an inflammatory process is triggered, NF-*κ*B binds to enhancers or superenhancers, triggering the transcription of enhancer RNA (eRNA), driving the chromatin of the NF-*κ*B-binding gene locus to construct transcriptional factories, and forming intra- or interchromosomal contacts. These processes reveal a mechanism in which intrachromosomal contacts appear to be *cis*-control enhancer-promoter communications, whereas interchromosomal regulatory elements construct *trans*-form relationships with genes on other chromosomes. This article will review emerging evidence on the genome organization hierarchy underlying the inflammatory response.

## 1. Introduction

Proinflammatory stimuli elicit transcriptional responses. In the inflammatory process, the multilevel response of NF-*κ*B contributes to the formation of cell-type specific and stimulatory responses. Thus, these NF-*κ*B responses can be divided into two parts: stimulated specific inflammatory responses and immune cell developmental responses [1–3]. These two responses are different but interrelated. In addition to differential functions of transcription factors (TFs), the transcriptional activity of proinflammatory genes is controlled by epigenetic markers, superenhancer (SE) dynamics, enhancer RNA (eRNA) transcription, transcriptional factories, chromatin structure, and intra- or interchromosomal contacts. Chromatin can contribute to the selectivity of NF-*κ*B responses, by providing not only the necessary barrier elimination for transcriptional activation but also the required chromatin environment for efficient gene expression.

Currently, the development of molecular biology technology has enabled the elucidation of molecular mechanisms underlying inflammation. Chromatin immunoprecipitation sequencing (ChIP-seq) can monitor dynamic changes in NF-*κ*B and other regulators of NF-*κ*B target genes at a genome-wide scale, whereas quantitative and complete analysis of transcriptomes through RNA sequencing (RNA-Seq) can be used to evaluate selective regulated subgroups of NF-*κ*B target genes [4]. Chromatin conformation capture (3C) technology is more effective in obtaining transcription factory-mediated dynamic information on intra- or interchromosomal contacts. In addition [5], global nuclear run-on sequencing (GRO-Seq) can be used to evaluate the regulation of eRNA within SEs in response to lipopolysaccharide (LPS) stimulation [6]. The application of these analytical techniques can be more beneficial in evaluating chromatin-dependent inflammatory responses.

## 2. Formation of Transcription Factories in Expression of Inflammatory Genes

Approximately 40 years ago, active genes were identified to be located at very close points of attachment [7]. A study reported that components of the transcriptional machinery cluster with each other and form factories; and that is two separated sites on a DNA loop can be combined together and two polymerases that are several kb apart on one template can come together spontaneously in the cellular nucleus [8]. The results obtained using the 3C technique have suggested that RNA transcription occurs in specialized sites termed as transcription factories [9–11]. Thus, transcriptional regulation is no longer considered to be a linear process. The genome is regulated by at least three levels, including local and long-range chromatin loops and interchromosomal contacts [12].

In the nucleus, RNA polymerase II transcription appears in a different dot-like spatial distribution, whereas transcriptional activity correlates with their localization within the transcription factories; however, equally inactive genes do not enter these factories. Adequate evidence suggests that proinflammatory responses are included in the transcription factories [13]. Most inflammatory genes before activation are irrelevant to transcription factories. However, following stimulation with LPS or TNF-*α*, these genes rapidly migrate to the transcription factories [10, 14]. Several studies have described the spatial dynamics of the way these factories work during TNF-*α* stimulation [15–17]. Furthermore, Fanucchi et al. reveal that cotranscription of the transcriptional factories is hierarchical, with dominant and subordinate members of the multigene complex engaged in both intra- and interchromosomal contacts [18], and such hierarchy might exist in response to TNF-*α* stimulation. The products of the transcriptional factories are rich in intronic-unprocessed transcript, long intergenic noncoding (lincRNAs), eRNAs, micro-RNA precursors, and repeat-derived RNAs [19]. Many studies have reported that the expression of inflammatory genes is associated with the transcriptional factories. In activated cells but not in resting human monocytes, stimulation with LPS triggered IL-1A, IL-1B, and IL-37 regulatory regions to move in close proximity, suggesting transcription by the same transcription factory [11]. Another study reported that synergistic responses of nascent mRNA and noncoding miRNA genes, including SAMD4A, EXT1, pre-mir-17, pre-mir-155, and pre-mir-191, to TNF-*α* stimulation are produced in active NF-*κ*B factories [10]. For nucleoplasmic factories, factory formation principles, factory specialization, and their regulation, Papantonis and Cook have a good discussion and a comprehensive review [20].

## 3. Chromatin Spatial Interactions in Expression of Inflammatory Genes

IFN-*γ* [21, 22], IL-4 [23–24], and IL-17 [25–27] are involved in various inflammatory responses. In response to antigen stimulation, naive T cells can differentiate into Th1, Th2, and Th17 cells expressing IFN-*γ*, IL-4, and IL-17, respectively. These cytokine genes are encoded on different chromosomes. The Th2 locus control region is crucial in the regulation of genes encoding the cytokines IL-4, IL-5, and IL-13, which are clustered in a 120-kilobase (kb) region in the mouse genome and a160 kb region in the human genome. In naive T cells, the Th2 locus control region participates and forms long-range interactions between the locus control region and promoters of IL-4, IL-5, and IL-13 genes [28, 29]. In addition to these intrachromosomal interactions, recent studies [30, 31] have reported interchromosomal interactions between the promoter region of IFN-*γ* gene on chromosome 10 or IL-17 gene on chromosome 1 and the regulatory regions of the Th2 cytokine locus on chromosome 11. The DNase I hypersensitive region at the Th2 locus developmentally regulates these interchromosomal interactions. This interaction between chromatin partners is a specific dynamic relationship of naive T cells; thus, interchromosomal contacts are apparently lost due to intrachromosomal clustering after gene activation [30, 31]. This presents a strategy to change the direction of naive T cell development to achieve the antagonism of chronic inflammation.

The TF NF-*κ*B plays a central role in inflammatory interchromosome contacts. During a viral infection, NF-*κ*B gets activated and mediates colocation of the IFN-*β* gene locus (9p21.3) with three distant NF-*κ*B-bound genomic loci, allele of number 21 (4p13), number 14 (9q33), and number 9 (18q21) [32]. Interchromosomal associations between the IFN-*β* locus and these loci appear before transcription initiation and during enhanceosome assembly (2–6 h) and are decreased at the time of initiation and propagation of transcription (6–8 h) [32]. In addition, a study reported that 11% of virus-infected cells colocalize between the IFN-*β* and IkBa gene locus (14q13.2) [5]. In the TNF gene locus, NF-*κ*B p50/p65 and NFATp binds to hypersensitive sites, which are 9 kb upstream (HSS−9) and 3 kb downstream (HSS+3) of the TNF gene. In HSS+3, NF-*κ*B p50/p65 only binds to binding sites NFAT-2840 with a high affinity and NFAT-2856 with lower affinity. By contrast, p50 and p65 subunits of NF-*κ*B are recruited to HSS−9 regions. Moreover, in HSS+3, p65 binding is strongly inducible, whereas p50 binding is slightly inducible [5]. In addition, in the process of macrophage activation, induced expression of microRNA- (miR-) 155 and miR-146a contributes to the regulation of inflammatory response and endotoxin tolerance [33–35]. In activated naive macrophages, NF-*κ*B p65 binds to miR-155 and miR-146a gene loci. DNA fluorescence in situ hybridization demonstrated monoallelic interchromosomal colocalization of miR-155 and miR-146a gene loci at the endotoxin tolerance stage, whereas RNA-DNA-fluorescence in situ hybridization indicated is silencing of the colocalized alleles, suggesting a common repression mechanism [36]. The highly transcribed housekeeping gene does not interact with any of the distal enhancers, while another 54% of the active promoters exhibit an extensive looping interaction with the enhancer and related to signal transduction pathways [37]. On the other hand, a confusing pre-existing chromatin-looping interactions at several loci are likely a common rule in different cell types. The study confirmed that p65 binding sites looping to the promoters prior to induction are much more likely to result in transcriptional activation of the linked gene than otherwise [37]. A similar study has also shown that NF-*κ*Β utilizes pre-existing chromatin looping to exert its multimodal role [38].

CTCF is a highly conserved zinc finger protein implicated in diverse regulatory functions, and many chromatin structures are involved in the insulator protein CTCF [39]. In recent years, genome-wide studies have provided adequate evidence that CTCF mediates intra- and interchromosomal contacts at several developmentally regulated genomic loci [40–42]. CTCF-mediated effects can be modulated by LPS or TNF-*α*. The cytokine genes TNF-*α*, lymphotoxin (LT) *α*, and LT*β* are regulated by NF-*κ*B signaling in inflammatory responses in the human TNF/LT gene locus. Watanabe et al. reported that along with TNF stimulation, CTCF insulators mediated intrachromosomal dynamic interactions between the enhancer and LT*α*/TNF promoters, followed by interaction with the LT*β* promoter, whereas CTCF depletion reduced TNF expression and accelerated LT*β* induction [43]. Similarly, LPS stimulation induced CTCF removal and noncoding RNA transcription on the −2.4 kb element of the chicken lysozyme locus in macrophages [44, 45]. However, CTCF-deficient macrophages manifest a strongly impaired capacity to produce TNF-*α* and IL-10 family members IL-10, IL-19, IL-20, and IL-24 upon toll-like receptor stimulation with LPS or R848 [46].

## 4. Regulation of SEs in Expression of Inflammatory Genes

Several studies have reported that enhancer-promoter and gene locus interactions indicate that genes driven by SE usually occur within the chromosome structure [31, 47]. SEs, also known as stretch enhancers, are a cluster of active enhancers having relatively long nucleotide sequence, high-density binding of TFs, and hypersensitivity to perturbation, which are different from typical enhancers (TEs). These SEs are clustered into a three-dimensional structure to regulate gene expression.

In 2013, Lovén et al., Whyte et al., and Hnisz et al. first reported about the SEs and their effects on diseases [48–50]. Nearly a year later, Brown et al. reported NF-*κ*B-induced SE formation in inflammation in atherosclerosis [51]. Furthermore, they reported that the DNA length of SE loci is longer than that of TE loci; that the absolute change in total signal and density of p65 and BRD4, a member of the bromodomain and extraterminal domain (BET) family, is higher following TNF-*α* stimulation; and that H3K27ac and BRD4 distribution in SE share less overlaps between resting and TNF-*α*-activated endothelial cells. More importantly, NF-*κ*B-formed SEs drive proinflammatory transcription and gene expression in a BET bromodomain-dependent manner [49]. A recent report indicated that senescence also involves a global remodeling of the enhancer landscape with recruitment of the chromatin reader BRD4 to newly activated SEs adjacent to key genes of senescence-associated secretory phenotypes (SASPs), such as IL-1*α*, IL-1*β*, and IL-8 [52]. Transcriptional profiling and functional studies have reported that BRD4 is required for SASP and downstream paracrine signaling [52]. Furthermore, a study reported that after LPS stimulation, genes that gain SE activity are basically involved in immune processes and inflammatory responses and genes that lose SE activity are often associated with cellular metabolism and nuclear organization functions [6]. In addition, NF-*κ*B-formed SEs drive proinflammatory microRNA gene expression [53].

TNF-*α*-activated SE target genes are different from SASP genes. These 62 TNF-*α*-gained SE target genes focus on cytokine signaling, chemotaxis, thrombosis, adhesion, and migration pathways, whereas 55 TNF-*α*-lost SE target genes are mainly in angiogenesis, antithrombotic, and barrier function [50]. Unlike TNF-*α*, 198 senescence-activated SE target SASP genes are based on cytokine activity, receptor binding, growth factor activity, and cytokine receptor binding pathways, whereas repressed 191 SASP genes are involved in sequence-specific DNA binding TF activity, nucleic acid binding TF activity, and fibronectin binding [51]. These findings indicate that disease-specific inflammation has different responses of SE activity and gene expression. Another example can clearly explain the disease specificity of SE. Peeters et al. [54] identify a disease-specific, inflammation-associated SE signature in synovial-fluid-derived CD4(+) memory/effector T cells of patients with juvenile idiopathic arthritis (JIA). They observed that the TFs ETS1 and RUNX1 had a higher binding enrichment in JIA-asssociated SEs, which could be distinguished from the classical NF-*κ*B-formed SEs. The genes regulated by JIA-asssociated SEs included chemokine and interleukin receptors, CD markers, and those associated with T cell activation and defense responses [54]. These findings provide a possible therapeutic approach for treatment of autoimmune diseases in the future.

p300 is a histone acetyltransferase that catalyzes hyperacetylation of histone H3 at multiple sites. The results of many studies support the notion that p300-marked SEs can help identify key nodes of transcriptional control during cell fate decisions [49, 55, 56]. SEs in TLR4-activated macrophages exhibit strong p300 binding and initiate gene transcription [55]. Vahedi et al. used ChIP-seq for the p300 protein and constructed SE catalogues of murine CD4^**+**^ Th1, Th2, and Th17 cells [56]. SEs with the highest p300 occupancy were typically associated with genes encoding cytokines and their receptors [56]. Furthermore, cytokine-related gene expression was connected to SEs in activated CD4^**+**^ T cells but not in nonimmune-related cells such as myotubes [56]. The highest p300 enrichment in SEs was associated with the Bach2 locus regardless of the lineage subset of CD4^**+**^ T cells. These results revealed that among 348 genes, 26% of those with the SE structure in CD4^**+**^ T cells were repressed by BACH2, and transcriptional upregulation at some of these domains correlated with the upregulation of nearby genes in Bach2-deficient cells, reflecting Bach2 as a key regulator of CD4^+^ T cell differentiation that prevents inflammatory diseases by maintaining the balance between tolerance and immunity [56]. The conclusion of the study on the effect of Bach2 is consistent with the results of two similar previous studies [57, 58].

Studies have reported TNF-*α* or LPS stimulation, or cell senescence, or host defense, H3K27 acetylation and BRD4 binding as one of the characteristics of inflammatory SEs [50, 51, 59], suggesting that SEs may be useful in revealing novel targets for treating inflammatory diseases.

## 5. Enhancer RNAs Are Involved in the Regulation of Inflammatory Gene Expression

Studies have confirmed the presence of enhancer RNAs [58, 59]. In macrophages activated by endotoxins, 70% of extragenic Pol_II peaks are associated with enhancers, and their transcription is frequently adjacent to inducible inflammatory genes, generating many fewer transcripts [60]. The kinetics of activation of these enhancer ncRNAs, relative to mRNA of downstream inflammatory genes, are very similar to each other, appearing at 30 min after stimulation, reaching maximal levels between 60 and 90 min, and then rapidly declining [60]. Moreover, enhancer transcription at these inducible gene loci displays a clear temporal pattern, in which upstream enhancer transcription precedes the induction of the downstream protein-coding gene [60]. IIott et al. [61] reported the response of 76 NF-*κ*B-regulated eRNAs to LPS. Similarly, such regulation is not only observed after LPS stimulation but also in H. pylori infection [62]. Another study reported that nearly 27% of transcripts in intergenic regions are classified as eRNAs, and only 30% of typical intergenic enhancers overlap with an eRNA, whereas nearly all SEs contain eRNAs within intergenic regions [63], implying eRNAs of all overlaying SEs are assembled into more active and functional enhancers. Caudron-Herger et al. [19] also identified numerous TNF*α*-responsive and NF-*κ*B-regulated factory eRNAs at intergenic and intragenic enhancers or HUVEC-specific SEs. IFN-*γ* priming activates an IRF1-dependent distal tumour necrosis factor/lymphotoxin (TNF/LT) locus element hHS-8 (8 kb upstream of the TNF transcription start site) and induces its eRNA synthesis [64], indicating that IFN-*γ* regulates TNF gene expression by chromatin remodeling and IRF1 recruitment prior to response to a secondary TLR4 stimulus in the TNF/LT locus.

Intergenic regions flanking LPS- or TNF*α*-inducible genes were observed to be enriched in BRD4 and H3K27Ac in multiple mouse and human cell types [50, 54, 59, 64, 65]. Furthermore, the acetylation of histone H3 sites K9, K14, and K27 and of histone H4 sites K5, K8, and K12 are correlated with active enhancers and promoters [66]. In particular, H4K5/8 ac is rapidly induced at TLR4-responsive promoters following treatment with TLR4 agonists, whereas changes in enhancer RNA expression and H4K8ac at pre-existing enhancers are highly correlated. Moreover, H4K8ac and H3K27ac exhibit a very strong correlation [66]. In addition, compared with stimulation by LPS alone, IFN-*γ* priming of THP-1 cells prior to LPS stimulation significantly enhances H3K27ac levels and eRNA synthesis [64]. Similarly, BRD4 regulates the transcription of eRNAs [67]. By contrast, the regulation of the expression of both enhancer lncRNAs and promoter lncRNAs is associated with H3K4me3 and H3K27Ac [65]. However, enhancer transcription precedes local H3K4 hypomethylation at de novo enhancers, which is primarily dependent on the histone methyltransferases Mll1, Mll2/4, and Mll3, and significantly reduced by inhibition of RNA polymerase II elongation [66]. In addition, eRNA-producing enhancers exhibited increased active mark H3K27Ac levels, decreased DNA methylation, and enriched DNA hydroxylase Tet1 [68].

In summary, in the inflammatory process, factors that regulate the expression of proinflammatory genes are regulated not only by NF-*κ*B transcription factor but also by hierarchies of epigenetic modifications and interchromosomal communication. Although NF-*κ*B has various functions in normal physiology and is not suitable as a therapeutic target, an in-depth understanding of the specificity of the NF-*κ*B response would be beneficial in strategies for individual NF-*κ*B target genes or select target gene subgroups as therapeutic targets.
